# Role of TOPK in lipopolysaccharide-induced breast cancer cell migration and invasion

**DOI:** 10.18632/oncotarget.15360

**Published:** 2017-02-15

**Authors:** Min-Ah Seol, Jung-Hwan Park, Ji Heun Jeong, Jungmook Lyu, Seung Yun Han, Sang-Muk Oh

**Affiliations:** ^1^ Department of Biochemistry, College of Medicine, Konyang University, Daejeon, Korea; ^2^ Department of Medical Science, Konyang University, Daejeon, Korea; ^3^ Department of Anatomy, College of Medicine, Konyang University, Daejeon, Korea

**Keywords:** TOPK, invasion, matrix metalloproteinase, breast cancer

## Abstract

Inflammation has been known to be linked to invasion or metastasis of breast cancer, which has poor prognosis, although the regulatory mechanism remains to be undiscovered. Here we show that T-LAK cell-originated protein kinase (TOPK) mediates pro-inflammatory endotoxin lipopolysaccharide (LPS)-induced breast cancer cell migration and invasion. The mRNA or protein level of TOPK, toll- like receptor4 (TLR4), interleukin (IL)-6, vascular endothelial growth factor (VEGF) or matrix metalloproteinase9 (MMP9) genes related to TLR4 signaling or tumor progression was induced by LPS treatment in MCF7 breast cancer cells, but the induction was abolished by stable knocking down of TOPK in MCF7 cells. Also, TOPK depletion decreased LPS-induced phosphorylation of p38, but not ERK and JNK among mitogen-activated protein kinases (MAPKs). On the other hand, we revealed that TOPK is essential for transcriptional activity of NF-κB or MMP9 promoter triggered by LPS. The induced promoter activity of NF-κB or MMP9 but not AP-1 was inhibited by knocking down of TOPK. Furthermore, we demonstrated that inhibitor of TOPK or MMP9 as well as MMP9 siRNA efficiently blocked LPS-induced migration or invasion of breast cancer cell lines. Interestingly, both of expression of TOPK and TLR4 were markedly increased in high-grade breast cancer. Collectively, we conclude that TOPK functions as a key mediator of LPS/TLR4-induced breast cancer cell migration and invasion through regulation of MMP9 expression or activity, implying a potential role of TOPK as a therapeutic target linking LPS-induced inflammation to breast cancer development.

## INTRODUCTION

Lipopolysaccharide (LPS) known as a trigger of inflammatory responses has been suggested to be implicated in several cancer invasion or angiogenesis [1–3]. Recent studies also indicated that toll-like receptors (TLRs) are expressed in many tumors and might play key roles in tumor progression. The role of TLR4, specific receptor for LPS, in tumor progression has been studied in various cancers involving colon, pancreas, liver and breast cancers [4–7]. Particularly, it has been shown that LPS/TLR4 has critical role in stimulation of invasiveness of breast cancer cells, and status of TLR4 expression is deeply linked to metastasis [6]. Breast cancer is known to be one of common cancers in women and metastatic breast cancer still retains poor prognosis. However, little is known about the underlying mechanism by which LPS/TLR4 regulates breast cancer progression involving migration, invasion or metastasis.

It has been shown that T-LAK cell-originated protein kinase (TOPK), serine/threonine mitogen-activated protein kinase (MAPK)-like protein kinase, was highly expressed in several cancer cells and closely implicated in tumorigenesis [8, 9]. Recently, TOPK has been suggested to act as a prognostic factor in some of cancers such as oral, ovarian, or lung cancer [10–12]. In particular, TOPK has been shown to play critical roles in proliferation or cell division of breast cancer cells [9, 13]. Also, it has been recently proposed that TOPK promotes prostate cancer invasion, which is an essential process for metastasis [14]. On the other hand, our previous study has demonstrated that TOPK activated by LPS/TLR4-induced signaling cascades leads to inducible nitric oxide synthase (iNOS) induction in leukemia cells, suggesting potential role of TOPK in LPS-mediated inflammatory response [15]. However, there is no knowledge about role of TOPK in LPS/TLR4-induced invasion of cancer, specifically breast cancer, cells.

Here we reveal that TOPK functions as an effector in breast cancer cell invasion induced by LPS/TLR4 signaling. We demonstrate that LPS induction of TOPK activity upregulates matrix metalloproteinase9 (MMP9) via NF-kB activation. Furthermore, depletion of endogenous TOPK expression or TOPK catalytic activity abrogated LPS-induced breast cancer cell migration or invasion. We provide evidence that TOPK might be a pivotal mediator in LPS/TLR4-mediated signal transduction pathways leading to breast cancer cell invasion.

## RESULTS

### LPS upregulates expression and activity of TOPK or TLR4 expression in MCF7 breast cancer cells

**Figure 1:**
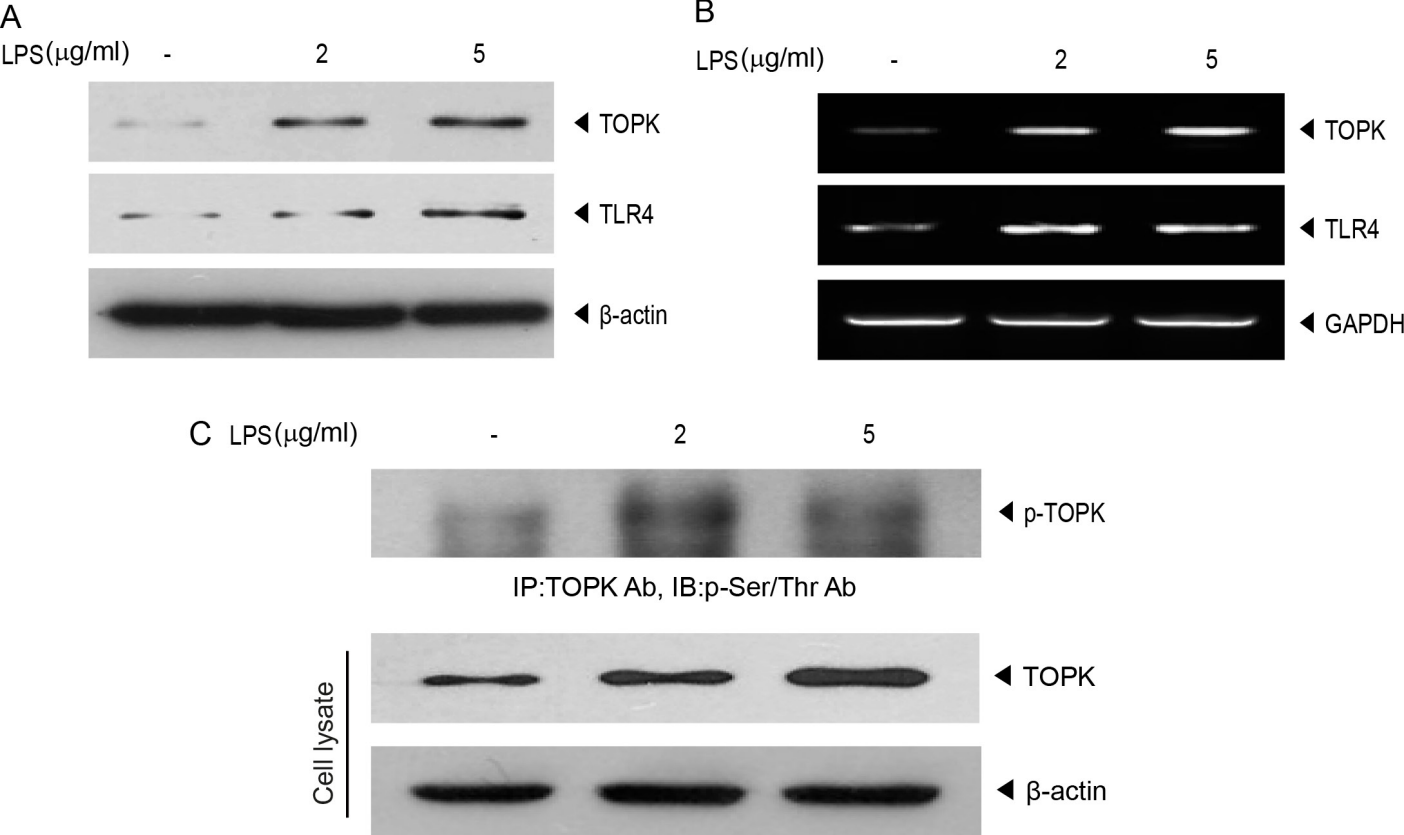
LPS promotes endogenous expression of TLR4 or TOPK, and TOPK phosphorylation in MCF7 cells MCF7 cells were treated with or without 10 μg/ml of LPS for 48 hr with indicated concentration. (**A**) Immunoblotting was done with TOPK or TLR4 antibody, and (**B**) RT-PCR was performed using each primer for TOPK, TLR4 or GAPDH genes. (**C**) Cell lysate was analyzed by immunoprecipitation with TOPK antibody and protein A/G plus-agarose bead, and then probed using phospho-serine/threonine (p-Ser/Thr) antibody. Endogenous TOPK level was verified using cell lysate. Representatives of three independent experiments are shown.

Based on our previous report [15] that LPS affected expression or activity of TOPK in immune cells, we investigated the effect of LPS on TOPK expression in MCF7 breast cancer cells. MCF7 cells were treated with LPS at concentration of 2 or 5 μg/ml. Results showed that protein or mRNA level of TOPK was increased dose-dependently in response to LPS in MCF7 cells, and TLR4, LPS specific receptor, expression was also elevated by LPS treatment (Figure 1A and 1B). Also, LPS markedly increased phosphorylation of serine and threonine (Ser/Thr) residues on TOPK (Figure 1C), implying LPS-induced TOPK activation. The augmentation of expression of TOPK as well as TLR4 in response to LPS in breast cancer cells suggests that TOPK might mediate LPS-TLR4 signaling leading to breast cancer cell migration. The proinflammatory stimuli, LPS binding to TLR4 was shown to cause breast cancer cell invasion [6].

### TOPK mediates LPS-induced breast cancer cell migration

**Figure 2:**
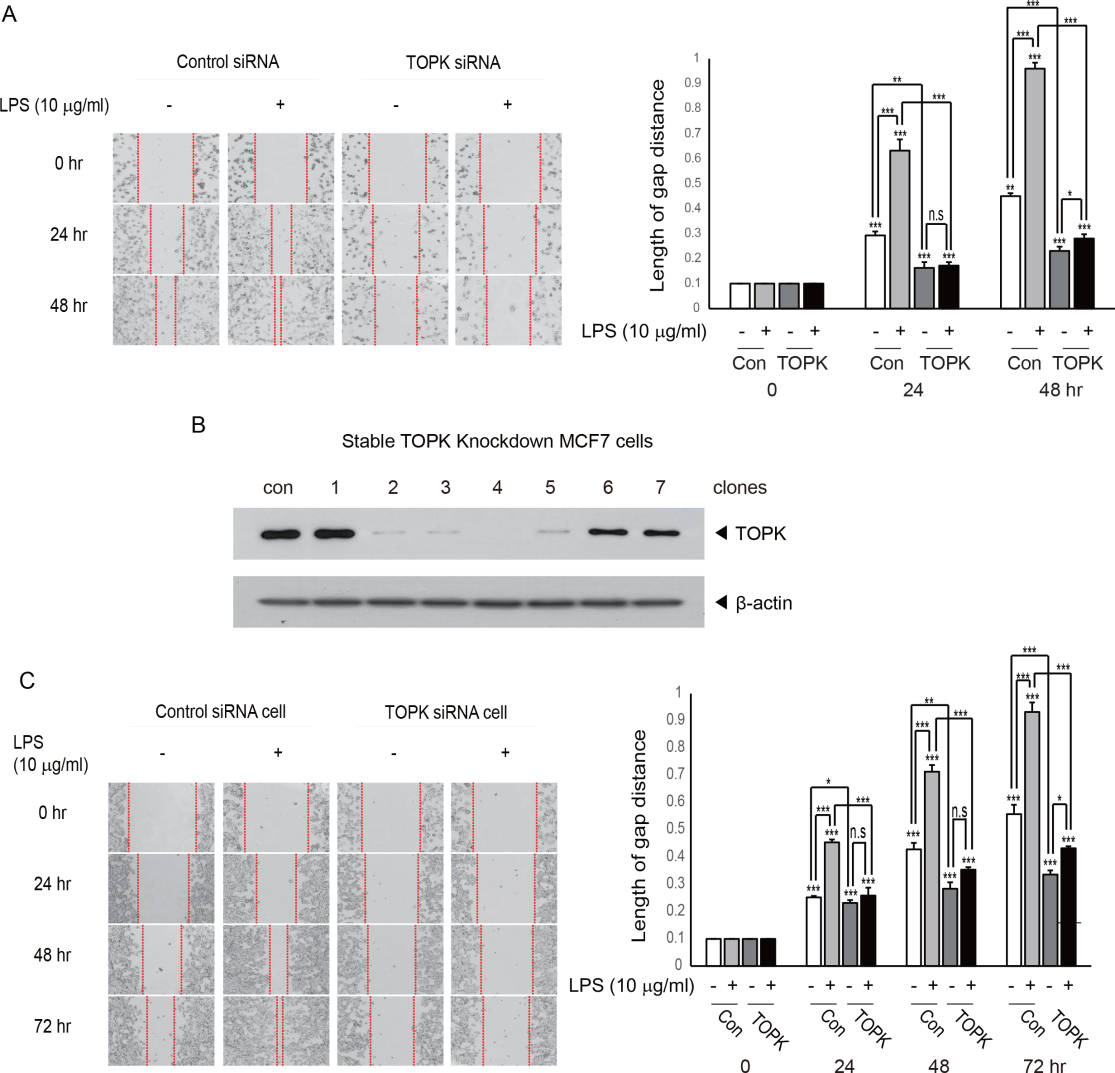
Knocking down of TOPK abrogates LPS-induced breast cancer cell migration (**A**) MDA-MB-231 cells were transfected with control siRNA (con) or TOPK siRNA (TOPK). 24 hr after transfection, each cell was incubated with or without LPS for indicated time, and then wound healing assay was done. (**B**) Stable transfectants of MCF7 cells were established with control siRNA or TOPK siRNA construct. The desired stable clone, control siRNA (con) or TOPK siRNA (clone 2) cells were selected, and used for further study. Endogenous expression of TOPK was examined. (**C**) Stable control siRNA (con) or TOPK siRNA (TOPK) cells were incubated with LPS for indicated time, and then cell migration was explored. Original magnification of images, x200. *, *p* < 0.05, **, *p <* 0.01, ***, *p <* 0.001 compared to controls. N.S, non-significant.

TOPK has been known to be involved in cancer cell growth. However, little is known about implication of TOPK in cancer cell invasion, which is a process essential for tumor metastasis. We next asked whether TOPK could mediate LPS-induced breast cancer cell migration. We investigated the effect of knocking down of TOPK on LPS-induced MDA-MB-231 breast cancer cell migration. MDA-MB-231 cells were transfected with control siRNA or TOPK siRNA. 24 hr after transfection, cells were treated with LPS (10 μg/ml) for indicated times. As shown in Figure 2A, transient expression of TOPK siRNA but not control siRNA markedly blocked LPS-induced cell migration time-dependently. To explore profound implication of TOPK in the breast cancer cell migration, we established stable MCF7 breast cancer cell line (TOPK siRNA cells) showing severely underexpressed TOPK using TOPK siRNA (Figure 2B). Using selected TOPK siRNA cells (clone 2) or control siRNA cells, we examined whether stable depletion of TOPK affected LPS-induced MCF7 cell migration. Results showed that LPS-induced migration of TOPK siRNA cell was greatly obstructed compared to that of control siRNA cell in time-dependent manner (Figure 2C). These findings suggest that TOPK can act as a critical mediator of LPS-induced breast cancer cell migration.

### Ablation of TOPK abolishes LPS-induced MMP9 expression, and reduces MAPK activation in MCF7 cells

**Figure 3:**
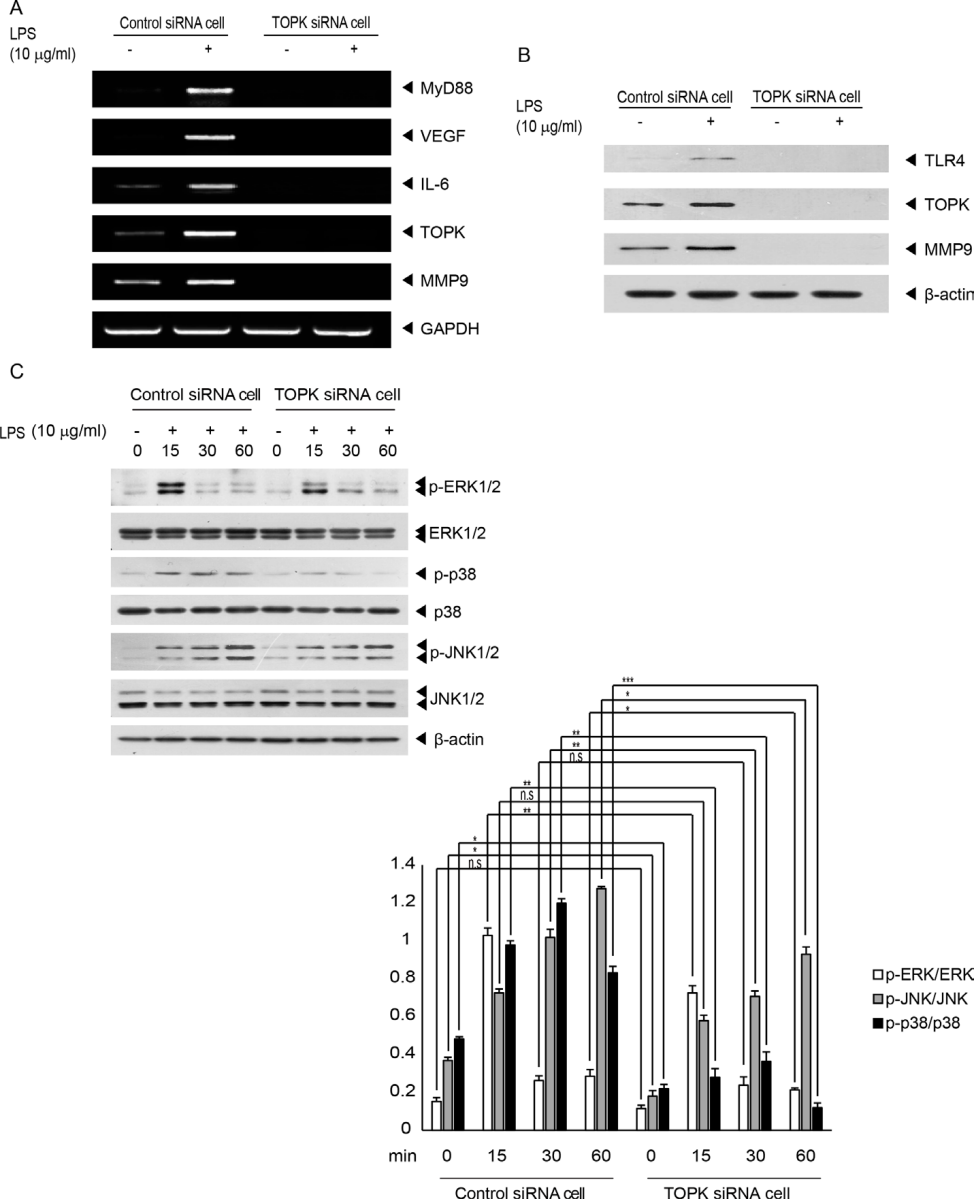
TOPK mediates LPS-induced endogenous expression of genes related to tumor progression or TLR4 signaling, and MAPKs activation stimulated by LPS Stable control siRNA cells or TOPK siRNA cells were incubated with or without LPS for 48 hr. (**A**) mRNA level for MyD88, VEGF, IL-6, TOPK, MMP9 or GAPDH genes was measured by RT-PCR using each primer. (**B**) Endogenous protein level of TLR4, TOPK, MMP9 or b-actin was evaluated by Immunoblot analysis with respective antibody. (**C**) Stable control siRNA or TOPK siRNA cells were stimulated with or without LPS for indicated times, and then probed with the indicated antibodies. Representatives of three independent experiments and graph for quantitation were shown. *, *p <* 0.05, **, *p <* 0.01, ***, *p <* 0.001 compared to controls. N.S, non-significant.

We next investigated the correlation of TOPK with genes related to angiogenesis, cell invasion or TLR4 signaling pathway, involving MMP9, vascular endothelial growth factor (VEGF), myeloid differentiation factor 88 (MyD88), or interleukin-6 (IL-6). Control siRNA cells or TOPK siRNA cells were treated with LPS (10 μg/ml) for 48 hr. LPS treatment of control siRNA cells but not TOPK siRNA cells resulted in increase of MMP9, VEGF, MyD88 and IL-6 as measured by RT-PCR (Figure 3A). Also, LPS-mediated MMP9 protein level was shown to be upregulated in control siRNA cells but not TOPK siRNA cells (Figure 3B). These data showed that TOPK might regulate expression of MMP9 critical for cell invasion. On the other hand, TOPK is known to belong to MAPKK-like protein kinase [16]. We next investigated whether depletion of TOPK affected LPS/TLR4 signaling cascades linked to MAPK. LPS (10 μg/ml) was added on control siRNA cells or TOPK siRNA cells for indicated times. Result showed that LPS-induced phosphorylation of p38, but not ERK and JNK among MAPKs was decreased in TOPK siRNA cells, compared to control siRNA cells (Figure 3C). These results demonstrated that TOPK could function as a key effector in LPS/TLR4 signal transduction involving MAPK activation leading to cancer cell migration or invasion.

### TOPK is required for LPS-induced MMP9 transcriptional activity in MCF7 cells

**Figure 4:**
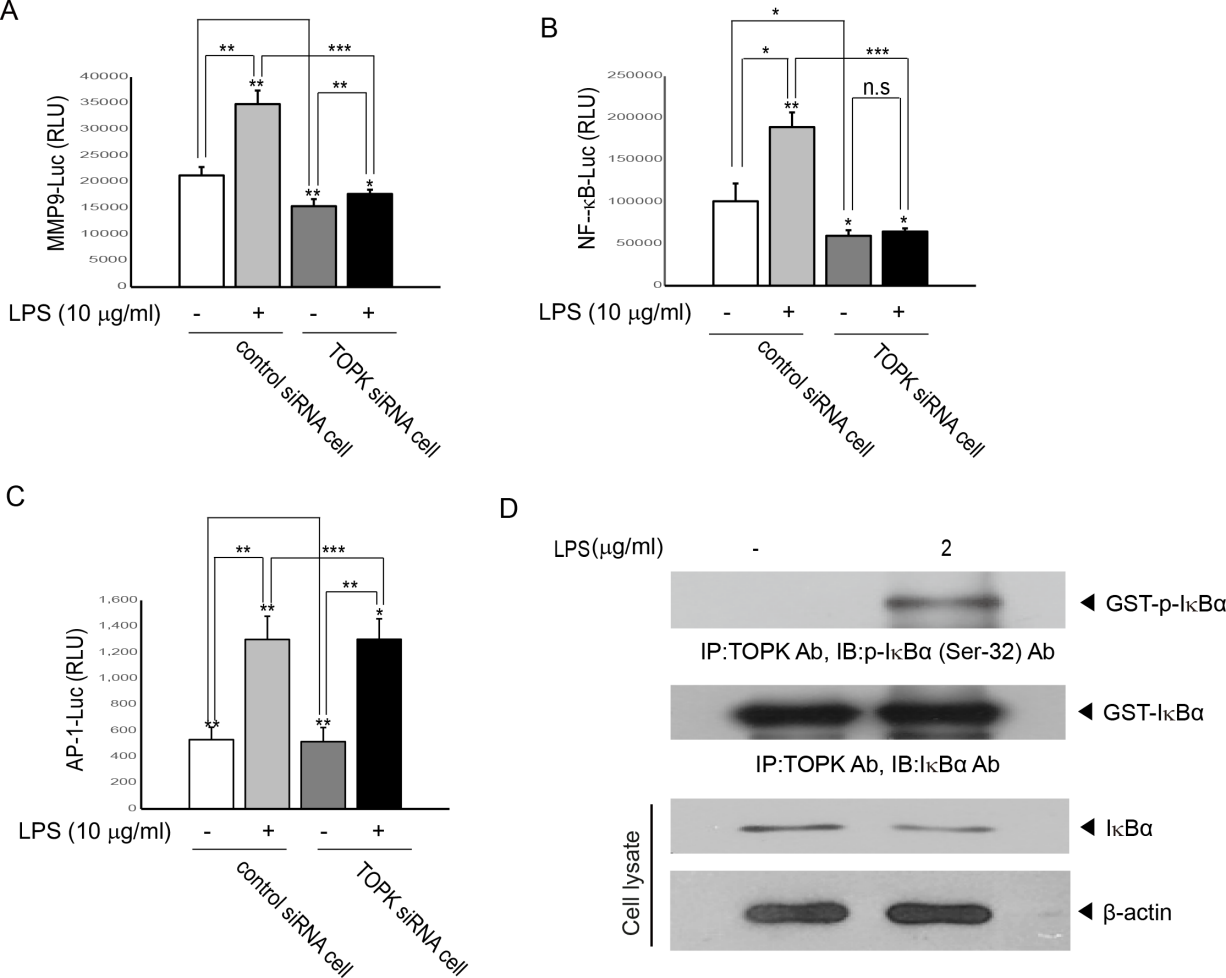
TOPK is essential for LPS-induced transcriptional activity driven by NF-kB- or MMP9- but not AP-1-promoter, and is activated by LPS Stable control siRNA cells or TOPK siRNA cells were transfected with (**A**) MMP9-, (**B**) NF-kB- or (**C**) AP-1-promoter linked to luciferase reporter constructs plus pRL-SV40 gene. 24 hr after transfection, cells were incubated with LPS for 48 hr. Firefly luciferase activity was normalized against Renilla luciferase activity. *, *p <* 0.05, **, *p <* 0.01, ***, *p <* 0.001 compared to controls. N.S, non-significant. (**D**) MCF7 cells were treated or not treated with LPS for 48 hr. Cell lysate was subjected to immunoprecipitation using TOPK antibody and protein A/G plus-agarose bead, and then kinase reaction was done using the recombinant GST-IkBa. The reactants were probed with p- IkBa antibody. Representatives of three independent experiments are shown.

We asked whether TOPK affected LPS-induced MMP9 promoter-driven transcriptional activity. Control siRNA cells or TOPK siRNA cells were transfected with MMP9 promoter-driven luciferase reporter construct, and then treated or not treated with LPS. As expected, LPS treatment increased MMP9 promoter-driven transcriptional activity in control siRNA cells, but not in TOPK siRNA cells (Figure 4A). Human MMP9 promoter is known to have functional cis-elements containing AP-1, NF-kB and Sp-1 elements [17]. We next investigated which transcription factor is involved in regulation of MMP9 promoter activity. Transcriptional activity of AP-1 or NF-kB, which are major transactivators for MMP9 promoter activity, was examined. AP-1 or NF-kB promoter construct linked to luciferase gene was expressed into control siRNA cells or TOPK siRNA cells, and then left in presence or absence of LPS. Results showed that knocking down of TOPK disrupted LPS-induced NF-kB promoter activity, but had no effect on AP-1 promoter activity (Figure 4B and 4C). Immunoprecipitation kinase assay also indicated that TOPK directly phosphorylated IkBa leading to NF-kB activity in MCF7 cells (Figure 4D). Collectively, these data suggest that TOPK positively regulates MMP9 expression through NF-kB activation in MCF7 cells.

### Depletion of MMP9 abolishes LPS-induced breast cancer cell migration

**Figure 5:**
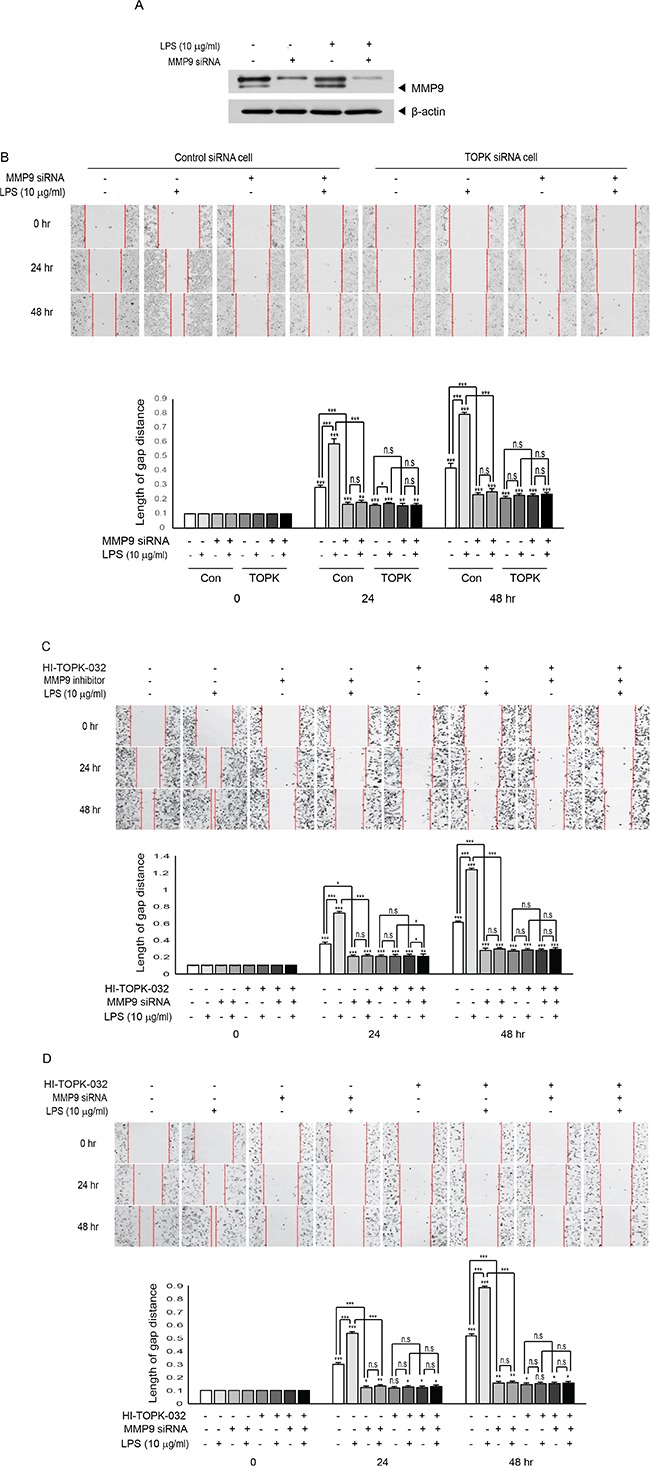
Suppression of MMP9 expression or activity abolishes TOPK-mediated breast cancer cell migration induced by LPS (**A**) MCF7 cells were transfected with control siRNA or MMP9 siRNA. 24 hr after transfection, cells were treated with or without LPS for 48 hr. Endogenous MMP9 expression was examined. (**B**) Stable control siRNA cells (Con) or TOPK siRNA cells (TOPK) were transfected with MMP9 siRNA. At 24 hr posttransfection, cells were treated or not treated with LPS for indicated times, and then migration assay was done. (**C**) MDA-MB-231 cells were treated with or without LPS in combinations of TOPK inhibitor, HI-TOPK-032 (10 mM) and MMP9 inhibitor (10 mM) for indicated times, and then cell migration was tested. (**D**) Hs 578T cells were transfected with control siRNA or MMP9 siRNA. 24 hr after transfection, cells were treated with or without LPS in absence or presence of HI-TOPK-032 (10 mM) for indicated times, and then cell migration was analyzed. Original magnification of images, x200. *, *p <* 0.05, **, *p <* 0.01, ***, *p <* 0.001 compared to controls. N.S, non-significant.

We next asked whether MMP9 indeed could exert its influence on MCF7 cell migration. Control siRNA cells or TOPK siRNA cells were transfected with MMP9 siRNA or control siRNA, and then treated or not treated with LPS. LPS treatment greatly induced migration of control siRNA cells but not TOPK siRNA cells. Also, expression of MMP9 siRNA dramatically reduced LPS-induced control siRNA cells migration, but had little effect on TOPK siRNA cells that already have little MMP9 expression (Figure 5A and 5B). We employed TOPK inhibitor, HI-TOPK-032 that is known to suppress TOPK catalytic activity to investigate effect of TOPK kinase activity on MDA-MB-231 cell migration. Also, MMP9 inhibitor was used to block MMP9 enzyme activity. MDA-MB-231 cells were treated with MMP9 inhibitor in presence or absence of HI-TOPK-032 or LPS. Suppression of TOPK activity resulted in remarkable decrease of LPS-induced MDA-MB-231 cell migration. Treatment with MMP9 inhibitor efficiently suppressed LPS-induced the cell migration (Figure 5C). To ensure these findings, we employed another invasive breast cancer cell line, Hs 578T cells were transfected with control siRNA or MMP9 siRNA, and then followed by treatment of combinations of HI-TOPK-032 or LPS. Result showed that cell migration was blocked by HI-TOPK-032 or MMP9 siRNA (Figure 5D), consistent with Figure 5C. Taken together, results demonstrated that MMP9 plays a key role in breast cancer cell migration, and that both of TOPK kinase activity and TOPK expression are required for LPS-induced MCF7 cell migration, suggesting the role of TOPK in regulation of MMP9 expression through NF-kB.

### Both of TOPK and MMP9 are essential for LPS-induced breast cancer cell invasion

**Figure 6:**
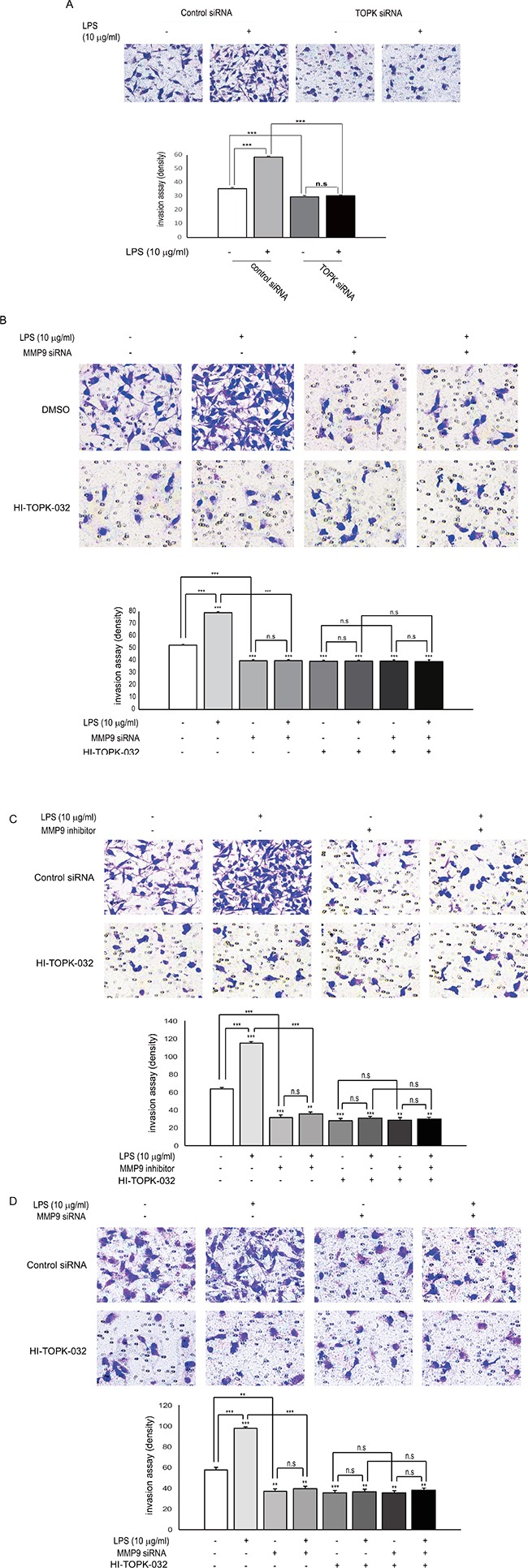
Both of expression or activity of MMP9 and TOPK are required for LPS-induced breast cancer cell invasion (**A**) MDA-MB-231 cells were transfected with control siRNA or TOPK siRNA. 24 hr after transfection, cells were treated with or without LPS for 48 hr, and then *in vitro* invasion assay was done. (**B**) MDA-MB-231 cells were transfected with control siRNA or TOPK siRNA. At 24 hr posttransfection, cells were treated or not treated with LPS in presence or absence of HI-TOPK-032 for 48 hr, and then invasion assay was performed. Representatives of at least three independent experiments are shown. (**C**) MDA-MB-231 cells were treated or not treated with LPS in combinations of TOPK inhibitor, HI-TOPK-032 (10 mM) and MMP9 inhibitor (10 mM) for indicated times, and then invasion assay was done. (**D**) Hs 578T cells were transfected with control siRNA or MMP9 siRNA. 24 hr after transfection, cells were treated with or without LPS in absence or presence of HI-TOPK-032 (10 mM) for indicated times, and then invasion assay was performed. **, *p <* 0.01, ***, *p <* 0.001 compared to controls. N.S, non-significant.

Tumor invasion is known to be a vital process because it is a pre-requisite for metastasis. Therefore, we next explored whether TOPK mediates LPS-induced breast cancer cell invasion. Control siRNA or TOPK siRNA was expressed into MDA-MB-231 cells in presence or absence of LPS. As expected, LPS treatment remarkably augmented MDA-MB-231 cell invasiveness. However, expression of TOPK siRNA substantially abrogated the cell invasiveness, compared with control siRNA (Figure 6A). Also, MDA-MB-231 cells were transfected with control siRNA or MMP9 siRNA, and incubated with LPS or HI-TOPK-032. LPS-induced invasiveness of MDA-MB-231 cells was markedly decreased by MMP9 siRNA expression, compared with control siRNA (Figure 6B). The invasiveness was also inhibited by HI-TOPK-032 treatment. Also, treatment with MMP9 inhibitor blocked LPS-induced invasiveness of MDA-MB-231 cells in absence or presence of HI-TOPK-032 (Figure 6C). Moreover, LPS-induced Hs 578T invasiveness was suppressed by MMP9 siRNA expression with or without HI-TOPK-032 (Figure 6D). These data demonstrate that both of TOPK and MMP9 might be key mediators of LPS-mediated breast cell invasion.

### Expression pattern of TOPK and TLR4 is similar and each expression is significantly elevated in high-grade breast cancer

**Figure 7:**
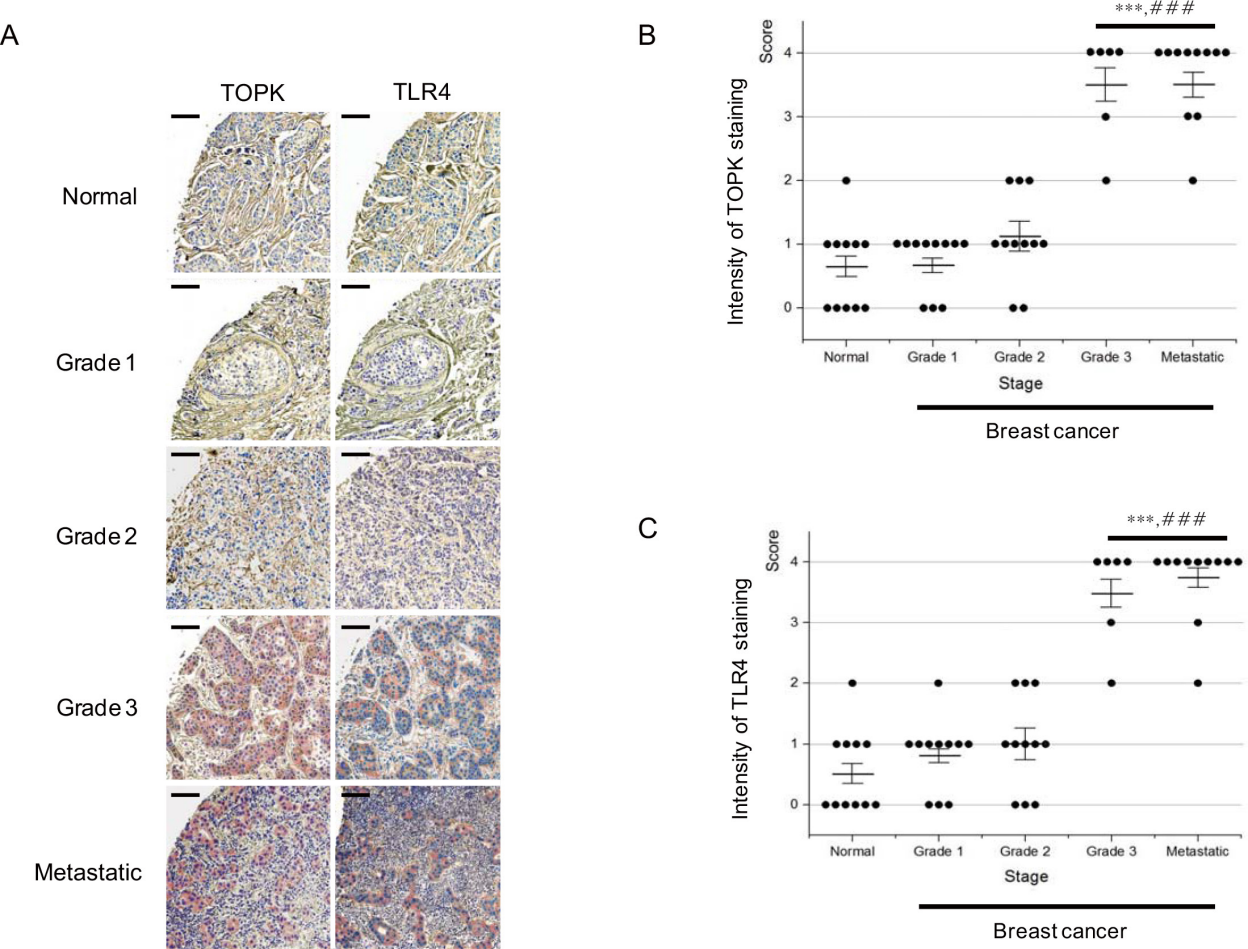
Expression of TOPK or TLR4 is highly increased in high-grade breast cancer (**A**) Representative immunohistochemistry images for detection of TOPK (left) and TLR4 (right) expression in human tissue array of normal breast ( *n* = 11), and breast cancer grade 1 (*n* = 11), 2 (*n* = 11), 3 (*n* = 6), and metastatic lymph node ( *n* = 11), respectively. Scale bar for all the images = 200 μm. (**B**) Intensity of TOPK staining was scored from 0 to 4, and then data were represented by dot plot. (**C**) Intensity of TLR4 staining was scored from 0 to 4, and then data were represented by dot plot. Average means of intensity and means±standard error of means (SEM) were also shown in each graph. P value is calculated by one way ANOVA: *** and ###, *P <* 0.001 vs. normal and grade 2, respectively.

It was shown that ablation of TOPK downregulated TLR4 expression (Figure 3). We next asked expression pattern of TOPK and TLR4 in breast cancers. Therefore, we performed immunohistochemistry using breast cancer tissue array paired with metastatic tumors. In total, the paraffin-embedded array was comprised of 12 cases of paired normal and primary, and 36 cases of primary and metastatic tumors in lymph node from the same patients in duplicates, 96 cores. In this study, due to tissue losses and damages resulting from antigen retrieval step, only 50 cores (*n* of tissues of normal, grade 1, 2, and metastatic = 11 and *n* of tissues of grade3 = 6) were processed for histologic grading. Immunohistochemical staining demonstrated that expression of TOPK or TLR4 was strongly correlated with breast cancer stage, and that expression pattern of TOPK or TLR4 was similar and significantly increased in high-grade breast cancer, invasive ductal carcinoma and lymph node metastasis (Figure 7 and Supplementary Table 1). These observations imply that both of TOPK and TLR4 might play a pivotal role in breast cancer invasion and metastasis.

## DISCUSSION

It has been suggested that inflammation might be one of symbols of cancer, and contributes to tumor cell proliferation, angiogenesis, metastasis, resistance to chemotherapeutic agents in tumor microenvironment [18]. The molecular pathways linking inflammation and cancer were being unraveled [19]. In the extrinsic pathway, inflammatory stimuli involving virus infection enhances cancer metastasis. Our study demonstrated that treatment of inflammatory LPS promoted TOPK expression and induced breast cancer cells migration or invasion, while TOPK ablation abolished the cell invasion, suggesting the potential role of TOPK in link of inflammation and breast cancer malignancy. Another pathway is related to genetic changes, which induce reconstruction of tumor microenvironment, resulting in expression of pro-tumorigenic factors involving IL-6, IL-8, VEGF, or MMP9. IL-6 was suggested to play a key role in development of breast cancer malignancy such as invasion and metastasis [20]. It has been proposed that MMP9, a 92 kDa type IV collagenase, exerts its ability as a key mediator of extracellular matrix (ECM) remodeling and metastasis through degradation of type IV and V collagens, and acts as prognostic biomarker for breast cancer patients [20]. Also, it has been suggested that VEGF and MMP-9 are critical cytokines for breast cancer cell invasion or metastasis, and that autocrine secretion of these cytokines by cancer cells importantly influences cancer cell behavior such as invasion [6]. We indicated that cytokines, IL-6, VEGF, or MMP9 were upregulated in response to LPS in MCF7 cells, but stable knocking down of TOPK abrogated the induced cytokines thereby influencing cell migration or invasion. These findings demonstrate that TOPK is required for LPS-mediated expression of these cytokines essential for tumor microenvironment leading to breast cancer cell invasion, implying TOPK is a requisite of breast cancer cell migration or invasion induced by LPS.

TLRs, a family of pattern recognition receptors, have been shown to be closely related to cancer progression. In particular, TLR4 expression was reported to have key roles in development of some tumors including prostate or ovarian cancers [22, 23]. A role of TLR4 in cancer progression might be somewhat controversial. It has been reported that activation of TLR4 on metastatic breast cancer cells stimulates invasiveness [24], whereas silencing of TLR4 enhances tumor progression and metastasis in a murine model of breast cancer [25]. However, LPS/TLR4-mediated regulatory mechanisms for breast cancer progression still remain elusive. In our study, it seems that treatment of LPS, a ligand of TLR4, increased TLR4 expression, thereby promoting MCF7 breast cancer cell migration or invasion. The expression of TOPK as well as TLR4 was augmented by LPS in MCF7 cells, but silencing of TOPK abolished the TLR4 expression, suggesting TOPK's regulatory role in TLR4 expression. We hypothesized that LPS/TLR4-induced TOPK expression or activity upregulates NF-κB activity, thereby augmenting TLR4 expression, and subsequent reinforcement of LPS-induced signaling. That is, there might be a positive feedback loop linking TLR4 and TOPK in LPS response. Our previous study has suggested that TOPK can directly activate NF-κB in response to LPS/TLR4 signaling in immune cells [15]. Also, it has been proposed that NF-κB activity might contribute to tumor progression and inhibition of NF-κB reduced breast cancer metastasis [26]. Furthermore, we showed that silencing of TOPK abrogated LPS-induced MMP9 promoter-driven transcriptional activity as well as MMP9 protein level, resulting in suppression of breast cancer cell migration or invasion in LPS/TLR4 signaling. Similarly, inhibition of TOPK catalytic activity using specific TOPK inhibitor HI-TOPK-032 suppressed LPS-mediated MDA-MB-231 breast cancer cell invasion. Possibly, these findings are due to loss of NF-κB activity induced by depletion of TOPK. On the other hand, TLR4 signaling was shown to be inhibited by intact ECM, which is abolished by MMP9-mediated ECM degradation, and agonists such as LPS are liberated [27]. We also hypothesized that LPS-mediated TOPK expression or activity leads to secretion of MMP9 thereby degrading ECM, which then activated TLR4 and enhanced breast cancer cells invasiveness. Significantly, immunohistochemical staining of TOPK or TLR4 on breast cancer tissues revealed that expression pattern of TOPK and TLR4 in each breast cancer stage was similar and both of them were significantly increased in high-grade breast cancers, suggesting the role of TOPK and TLR4 in breast cancer metastasis. It appears that elevation of TLR4 expression by TOPK in LPS response might occur via NF-kB or MMP9-mediated positive feedback loop leading to breast cancer invasion and metastasis, suggesting a possible mechanism regarding TOPK-mediated regulation of TLR4.

In conclusion, our study demonstrates that TOPK is a key mediator in LPS/TLR4 signaling to trigger breast cancer invasion, and that LPS/TLR4 signaling enhances expression and activity of TOPK leading to NF-κB activation, thereby enhancing MMP9 expression and subsequent breast cancer cell invasion. TOPK-mediated regulation of NF-κB activity and MMP9 expression in LPS/TLR4 signaling might be a mechanistic link connecting inflammation to breast cancer malignancy. Further studies for LPS/TLR4-TOPK signaling cascades are required for elucidation of molecular mechanism leading to breast cancer metastasis.

## MATERIALS AND METHODS

### Cells and reagents

Human breast adenocarcinoma cell lines, MCF7 cells and MDA-MB-231 cells were purchased from American Type Culture Collection (ATCC). Each cell line was maintained in DMEM supplemented with 10% fetal bovine serum (FBS), 2 mM L-glutamine, and 1% penicillin/streptomycin. Transfection reagent, Effectene or lipofectamine 3000 was from Qiagen, Inc. (Valencia, CA) or Invitrogen (Grand Island, NY), respectively. ON-TARGETplus SMARTpool MMP9 siRNA and siGENOME Non-targeting control siRNA was from GE Dharmacon (Lafayette, CO). Lipopolysaccharide and b-actin antibody was purchased from Sigma (St. Louis, MO). Antibodies against p-p38 (Thr180/Tyr182), p-JNK (Thr183/Tyr185), p-ERK1/2 (Thr202/Tyr204) and p-IkBa (Ser-32) were from Cell Signaling Technology, Inc. (Beverly, MA). Antibodies against TOPK, TLR4, MMP9 or p-serine/threonine, and breast cancer tissue array paired with metastatic tumors or MMP9 inhibitor were purchased from Abcam (Cambridge, MA). Luciferase assay system was from Promega (Madison, WI). IkBa antibody or protein A/G plus-agarose bead was purchased from Santa Cruz Biotechnology, Inc. (Santa Cruz, CA). TOPK inhibitor, HI-TOPK-032 was from R&D system (Minneapolis, MN). SuperScript III reverse transcriptase was from Invitrogen (Grand Island, NY). GST-IkBa protein was from EMD Millipore (Billerica, MA).

### Plasmids and construction of stable TOPK siRNA cells

NF-kB promoter or MMP9 promoter-driven luciferase constructs were as described, respectively [28, 29]. AP-1 promoter-luciferase reporter vector was from Addgene. MCF7 cell line stably expressing TOPK siRNA or control siRNA was established as described previously [28]. Briefly, MCF7 cells growing on 100 mm dish were transfected with 4 μg of TOPK siRNA or control siRNA construct using effectene reagent. At 24 hr posttransfection, cells were changed with G418-containing media. After about two months, the desired clone was selected.

### Immunoblot and immunoprecipitation

20 μg of cell lysate was separated on 10% SDS-PAGE, and then immunoblot was done using each respective antibody, and the blot was analyzed with SuperSignal west pico chemiluminescent substrate (Pierce Biotechnology, Inc., Rockford, IL) and X-ray film. Immunoprecipitation was performed using TOPK antibody and protein agarose beads. Each antibody and bead was added to 500 μg of LPS-treated cell lysate, and incubated at 4°C for 2 hr using rocker. The beads were washed, and subjected to 10% SDS-PAGE, and subsequent immunoblot analysis.

### *In vitro* kinase assay

MCF7 cells were treated with LPS, and cell lysate was incubated with TOPK antibody and beads. Kinase assay was carried out using the immunoprecipitates and GST-IkBa protein at 30°C for 30 min. The beads were subjected to 10% SDS-PAGE and immunoblotting using phospho-IkBa (Ser-32) antibody.

### Luciferase assay

Stable TOPK siRNA cells or control siRNA cells growing on six well plates were co-transfected with each 1 μg of MMP9, AP-1, or NF-kB promoter-luciferase reporter constructs plus 0.5 μg of the *pRL-SV40* gene. 24 hr after transfection, cells were treated with lipopolysaccharide for 48 hr. Firefly and Renilla luciferase activities were estimated using cell lysate.

### Reverse transcription-PCR

Total RNAs were prepared from stable control siRNA cells or TOPK siRNA cells treated with or without LPS using easy blue reagent (Introen). Reverse transcription-PCR was perfomed using SuperScript III reverse transcriptase (Invitrogen) and PCR master mix (Qiagen). PCR was done for 1 cycle at 95°C for 15 min, and 28 cycles at 95°C for 30 s, 55°C for 30 s, and 68°C for 1 min. Each primer is as follows. TOPK (forward), 5'-TCCTGCCTTCATAACCATCTT-3'; TOPK (reverse), 5'-ATTCTCCTCCACAGCTTCTT T-3'; TLR4 (forward), 5'-CAAGAACCTGGACCTGAGCT-3’; TL R4 (reverse), 5'-ATTGCACAGGC CCTCTAGAG -3'; MyD88 (forward), 5'-CGGATGGTGGTGGTTGTCTC -3'; MyD88 (reverse), 5'-CGCTTCTGATGGGCACCT -3'; IL-6 (forward), 5'- ATGAACTCCTTCTCCACAAG C-3'; IL-6 (reverse), -CTACATTTGCCGAAG AGCCCTCAGGCTGGACTG-3'; VEGF (forward), 5'- ATCTGCA TGGTGATGTTGGA-3'; VEGF (reverse), 5'- GGGCAGAATCATCACGAAGT-3'; MMP9 (forward), 5'- TGTACCGCTATGGTTACAC-3'; MMP9 (reverse), 5'-CCGCGACACCAAACTGGAT-3'. PCR products were subjected to agarose gel electrophoresis and analyzed with GelDoc (Biorad).

### Wound healing assay

Breast cancer cell migration was explored using a wound-healing assay. 5 × 10^5^ of MCF7 cells or 3 × 10^5^ of MDA-MB-231 or Hs 578T cells was seeded at six well plates. Center area of each cell was scratched with a sterile 100 μ*l* pipette tip, and washed with PBS. The medium was changed and cells were allowed to migrate. Micrograph images were taken with a microscope at the indicated time points following LPS treatment.

### *In vitro* invasion assay

*In vitro* invasion assay was done using invasion assay kit with Matrigel-coated inserts (BD Biosciences, San Jose, CA). Each 1 × 10^5^ cells/well was seeded at upper compartment of the invasion chamber, and serum-free conditioned medium was added at lower compartment in presence or absence of LPS, MMP9 inhibitor or HI-TOPK-032. The cells of upper chamber were allowed to invade to the lower surface of the collagen coated membrane toward the lower chamber. The filters were fixed and stained with Diff-Quik reagents (Dade Behring, Inc., Newark, DE).

### Immunohistochemistry

Breast cancer tissue array paired with metastatic tumors was used for immunostaining of TOPK and TLR4. In brief, after deparaffinization and subsequent 12 hr incubation with rabbit polyclonal antibody for TOPK(1:200) and TLR4 (1:200), biotinylated anti-rabbit IgG (1:200) diluted in phosphate-buffered saline were applied as secondary antibody. After incubation with alkaline phosphatase substrate solution, counterstaining with hematoxylin was followed. The degree of immunostaining was evaluated by independent observer who was blind to the clinical stages. Cytoplasmic or membrane-bounded expression of TOPK and TLR4 were analyzed by the intensity of positive cells using Image J software (http://rsb.info.nih.gov/ij) and was ranked on an overall scale from 0 to 4 as described [30]; with 0 indicating the absence of staining; 1, weak staining; 2, moderate staining; 3, strong staining; and 4, highest staining.

### Statistical analysis

Results are shown as the mean ± SD for at least three independent experiments in duplicates. Significant differences were evaluated by student's *t* test or one way ANOVA.

## SUPPLEMENTARY MATERIALS TABLE




