# Supplementary material for: Role of TOPK in lipopolysaccharide-induced breast cancer cell migration and invasion
Source: Oncotarget. 2017 Feb 15;8(25):40190–203. doi: 10.18632/oncotarget.15360 (PMC5522254; doi:10.18632/oncotarget.15360)
Supplement: Supplementary file 1 [file oncotarget-08-40190-s001.pdf]

Role of TOPK in lipopolysaccharide-induced breast cancer cell migration and invasion

Supplementary Materials

Supplementary Table 1: Patient information. See Supplementary_Table_1
