# Supplementary material for: Role of TOPK in lipopolysaccharide-induced breast cancer cell migration and invasion
Source: Oncotarget. 2017 Feb 15;8(25):40190–203. doi: 10.18632/oncotarget.15360 (PMC5522254; doi:10.18632/oncotarget.15360)
Supplement: Supplementary file 2 [file oncotarget-08-40190-s002.docx]

patient information

| Array  Position | Age | Anatomic  Site | Nature | Pathology | Grade |
| --- | --- | --- | --- | --- | --- |
| B01 | 29 | breast | normal | - | - |
| B03 | 80 | breast | normal | - | - |
| B04 | 45 | breast | normal | - | - |
| B05 | 36 | breast | normal | - | - |
| B06 | 35 | breast | normal | - | - |
| B07 | 57 | breast | normal | - | - |
| B08 | 52 | breast | normal | - | - |
| B09 | 38 | breast | normal | - | - |
| B10 | 51 | breast | normal | - | - |
| B11 | 43 | breast | normal | - | - |
| B12 | 38 | breast | normal | - | - |
| C07 | 43 | breast | malignant | Invasive lobular carcinoma | 1 |
| C08 | 54 | breast | malignant | Invasive lobular carcinoma | 1 |
| C11 | 46 | breast | malignant | Invasive ductal carcinoma | 1 |
| C12 | 39 | breast | malignant | Invasive ductal carcinoma | 1 |
| A03 | 80 | breast | malignant | Invasive ductal carcinoma | 1 |
| E01 | 35 | breast | malignant | Invasive lobular carcinoma | 1 |
| E03 | 35 | breast | malignant | Invasive ductal carcinoma | 1 |
| E04 | 39 | breast | malignant | Invasive ductal carcinoma | 1 |
| E09 | 39 | breast | malignant | Invasive lobular carcinoma | 1 |
| E11 | 47 | breast | malignant | Invasive lobular carcinoma | 1 |
| G10 | 50 | breast | malignant | Invasive ductal carcinoma | 1 |
| A06 | 35 | breast | malignant | Invasive ductal carcinoma | 2 |
| A07 | 57 | breast | malignant | Invasive ductal carcinoma | 2 |
| A08 | 52 | breast | malignant | Invasive ductal carcinoma | 2 |
| A09 | 38 | breast | malignant | Invasive ductal carcinoma | 2 |
| C03 | 47 | breast | malignant | Invasive ductal carcinoma | 2 |
| C04 | 50 | breast | malignant | Invasive ductal carcinoma | 2 |
| C05 | 52 | breast | malignant | Invasive ductal carcinoma | 2 |
| E06 | 44 | breast | malignant | Invasive ductal carcinoma | 2 |
| E07 | 55 | breast | malignant | Invasive ductal carcinoma | 2 |
| E08 | 30 | breast | malignant | Invasive lobular carcinoma | 2 |
| E12 | 50 | breast | malignant | Invasive ductal carcinoma | 2 |
| A01 | 29 | breast | malignant | Invasive ductal carcinoma | 3 |
| A05 | 36 | breast | malignant | Invasive ductal carcinoma | 3 |
| C01 | 63 | breast | malignant | Invasive ductal carcinoma | 3 |
| C02 | 60 | breast | malignant | Invasive ductal carcinoma | 3 |
| C06 | 54 | breast | malignant | Invasive ductal carcinoma | 3 |
| E05 | 50 | breast | malignant | Invasive ductal carcinoma | 3 |
| F01 | 35 | lymph node | malignant | Lymph node metastasis | metastatic |
| F02 | 40 | lymph node | malignant | Lymph node metastasis | metastatic |
| F03 | 35 | lymph node | malignant | Lymph node metastasis | metastatic |
| F04 | 39 | lymph node | malignant | Lymph node metastasis | metastatic |
| F05 | 50 | lymph node | malignant | Lymph node metastasis | metastatic |
| F06 | 44 | lymph node | malignant | Lymph node metastasis | metastatic |
| F07 | 55 | lymph node | malignant | Lymph node metastasis | metastatic |
| F08 | 30 | lymph node | malignant | Lymph node metastasis | metastatic |
| F09 | 39 | lymph node | malignant | Lymph node metastasis | metastatic |
| F10 | 41 | lymph node | malignant | Lymph node metastasis | metastatic |
| F11 | 47 | lymph node | malignant | Lymph node metastasis | metastatic |
